# Implementation of analytical technologies in a pharmaceutical development organization looking into the next millennium

**DOI:** 10.1155/S1463924600000055

**Published:** 2000

**Authors:** Nigel North

**Affiliations:** Pharmaceutical Technologies SmithKline Beecham Pharmaceuticals Essex Harlow UK

## Abstract

Managing the implementation of new technology in a pharmaceutical development environment has provided challenges and opportunities to obtain benefits from technologies, e.g. laboratory automation. Successful application of new techniques requires a dedicated resource. Within Pharmaceutical Technologies, this was initially a single person, who has since evolved into a team dedicated to the investigation and development of robotics and non-invasive analytical techniques. Pharmaceutical development is an important interface between research and commercial manufacturing. In research, the success of genomics and combinatorial chemistry will result in a significant increase in the number of development compounds, and this, combined with the desire of commercial manufacturing to move towards parametric release, puts an emphasis on the need for rapid analytical methods. Some ideas on the techniques that will be required to meet these goals will be described together with their impact on automation.


Journal of Automated Methods & Management in Chemistry, Vol. 22, No. 2 (March–April 2000) pp. 41–45

### Implementation of analytical technologies in a pharmaceutical development organization—looking into the next millennium

**Nigel North**

*Pharmaceutical Technologies, SmithKline Beecham Pharmaceuticals, Harlow, Essex, UK*

*Managing the implementation of new technology in a pharmaceutical development environment has provided challenges and opportunities to obtain benefits from technologies, e.g. laboratory automation. Successful application of new techniques requires a dedicated resource. Within Pharmaceutical Technologies, this was initially a single person, who has since evolved into a team dedicated to the investigation and development of robotics and non-invasive analytical techniques. Pharmaceutical development is an important interface between research and commercial manufacturing. In research, the success of genomics and combinatorial chemistry will result in a significant increase in the number of development compounds, and this, combined with the desire of commercial manufacturing to move towards parametric release, puts an emphasis on the need for rapid analytical methods. Some ideas on the techniques that will be required to meet these goals will be described together with their impact on automation.*

### Introduction

In research, platform technologies including DNA sequencing, combinatorial chemistry and high-throughput screening (HTS) have increased the productivity in compound and lead generation. HTS can now provide the capability of screening 100 000 compounds per week; this will soon increase to 100 000 compounds per day. Combinatorial chemistry is rapidly developing to provide the libraries of compounds to keep up with the screening capacity and to increase the quality of development candidates. This capability has resulted in SB working on some 200 drug targets in 1997, which is nearly half of the 500 targets available to the entire pharmaceutical industry up until 1995. The supply of compounds into pre-clinical development will therefore see a significant increase in the future. The effect of this potential increase in development compounds on the pre-clinical organization in terms of the development of automation and other new technologies will be discussed with particular emphasis on managing its implementation in pharmaceutical development.

### Discussion

### *Development of new technologies in pre-clinical development*

The departments in pre-clinical development are introducing technologies to improve their efficiency. In chemical development, automation is being employed to help optimize the reaction steps during scale-up using equipment developed in Europe by Anachem Ltd. This equipment comprises a multiple-reaction block (STEM corporation), Gilson 233XL autosampler and an HPLC system. Spectroscopic methods including infrared are also being employed to monitor reactions on-line in process chemistry providing more rapid feedback on the progress of the reactions compared to traditional separation methods. Chromatography for drug substance purity determination and purification is being performed using parallel HPLC columns. The equipment supplied by Biotage is a typical example of this concept. Fast-gradient HPLC using short columns (2 mm ID × 10 mm in length) is also being applied to reaction monitoring enabling run times to be reduced to ~1 min.

In the analysis of biofluids, the implementation of LC/MS technology has increased productivity markedly with analysis times typically less than 5 min. The chromatography in this case is used more as a means of sample introduction, and the 'separation' is performed by the mass spectrometer. The requirement for high-speed sample introduction for LC/MS technology has resulted in the development of faster autosamplers for serial sample introduction. More recently, multi-line autosamplers have been developed to increase sample throughput into mass spectrometers.

The techniques described above are by no means an exhaustive account of the analytical technologies being developed to increase the efficiency of some areas in pre-clinical development; however, this does provide an overview of some of the trends in future technologies. The main themes that are evident include: automation; rapid separations; parallel analytical processing; fast sensitive and selective detectors; and non-invasive analysis.

These developments will improve the rate of compound supply, which in turn will impact the clinical supplies operation. In drug product analysis, well-established automation systems for tablet processing and dissolution of solid oral dosage forms have been implemented. These technologies, however, will not be sufficient to keep up with the increase in development candidates from research or with the emerging alternative technologies that will compete with the current analytical methods.

### *Managing the implementation of new analytical techniques in Pharmaceutical Technologies (UK)*

Pharmaceutical Technologies is responsible for the formulation and analytical development, clinical trials supply and technology transfer of new products to manufacturing facilities. Within Pharmaceutical Technologies (UK), the effort devoted to developing and implementing analytical techniques including automated methods has gradually increased over the past 6 years. Initially a single person was assigned to developing and validating new automated methods using the Zymate™ dissolution robot and the Benchmate™ within Analytical Development. In 1992, the department was reorganized into a multidisciplinary team-based structure. This resulted in the traditional analytical and formulation departments being reorganized into 'product' teams comprising formulators and analysts together with several specialist 'support' teams that perform tasks, e.g. microbiology and automated methods (Analytical Support). A central team to develop and validate was considered to be most effective for several reasons. (i) The experience gained from performing this process could be built up in the Analytical Support team and then shared with the Product teams on a timely basis enabling faster implementation of automated methods. (ii) Qualification and change control of robotic systems is easier to perform using a central team. (iii) Consistent approaches to validation of automated methods are more likely when the same scientists are involved in the process.

The reorganization of the department into this team-based structure also resulted in the Analytical Support team facing a significant change in the interfaces and provided communication challenges. The team has moved from being in a department in which all the analytical work was concentrated to interfacing with more than 10 individual teams with projects at various stages of development and each progressing at different speeds. Although establishing the initial outline for each project was time consuming, keeping the information up to date is relatively easy. The information typically recorded is shown below.

(1) Compound.
(2) Priority.
(3) Qualification batch manufacturing date.
(4) Status of automated method for:
    TPWII™
    Powder
    Bulk assay
    Content uniformity
    Degradation profile
    MultiDose™ dissolution.
(5) Product team.

In 1997, Pharmaceutical Technologies (UK) was consolidated from three different sites into a single building at Harlow, UK. During the past 18 months, the resource and remit of this team involved with new analytical technologies has now been widened to cover the following.

(1) Implementation of automated methods (three scientists).
(2) Development of non-invasive techniques, e.g. near-infrared and ultrasound (two scientists).
(3) Investigation of *in vitro* 'dissolution' systems for improving the prediction of *in vivo* performance of poorly soluble drugs (one scientist).

To drive the development of new technology (e.g. non-invasive analytical methods) forward faster in the UK, collaborations have been set up with groups external to

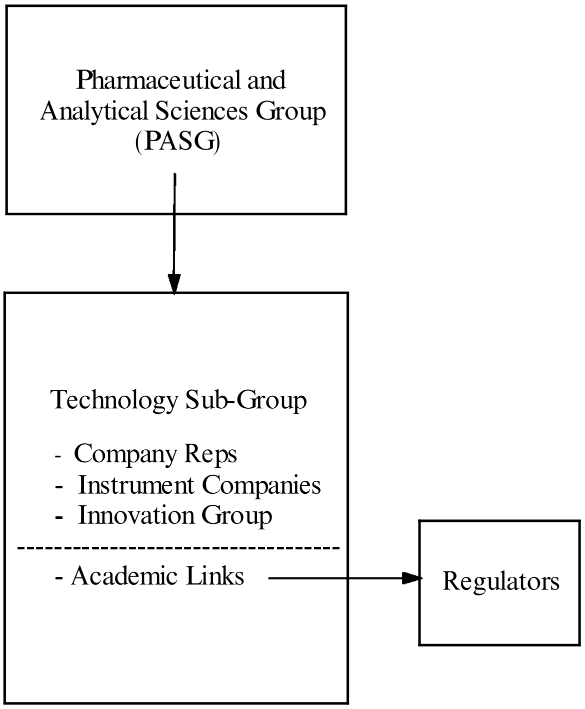

*Figure 1. PASG technology development process.*

SB. There are several reasons for these links. Firstly, to increase the level of resource devoted to new technology development without increasing the headcount. Secondly, to promote the development and regulatory acceptance of non-invasive technologies, e.g. near-infrared and more recently ultrasound. An example of this scenario is collaborations coordinated by the Pharmaceutical and Analytical Sciences Group (PASG) in the UK (figure 1). This group represents the major research-based pharmaceutical companies in the UK with the main aims of: (i) providing a unified voice on analytical issues via the Association of British Pharmaceutical Industry to international regulatory agencies; (ii) enhancing awareness of analytical science in education; and (iii) promoting the image and profession of pharmaceutical analysis. The PASG has sponsored the setting up of the Near Infrared Centre of Excellence at the London School of Pharmacy by supporting four PhD students. The Centre has been working in four key areas in which the regulators have concerns to aid the acceptance of the technology. Collaboration has also just been initiated on the application of ultrasound technology to pharmaceutical analysis.

### *Development of new technologies in pharmaceutical analysis*

Automation of the analysis of pharmaceutical drug products over the past 30 years has resulted in relatively small stepwise improvements in efficiency. In the 1970s, automation of traditional colorimetric assays for drugs, e.g. penicillins was achieved by the use of autoanalysers. This was followed by the advent of HPLC, and in the early 1980s unattended analysis was implemented utilizing autosamplers. Dissolution robots became available in the mid-1980s followed by the tablet processing systems in the mid-1990s. These technologies have helped improve efficiency in sample throughput but these are unlikely to be sufficient to keep pace with the developments in research and the needs of commercial manufacturing.

The weaknesses of current pharmaceutical analytical processes will be outlined to enable better understanding of how appropriate newer technologies will be used in our laboratories in the future. A generic process for drug product analysis is outlined in figure 2.

(1) *Request for analysis.* In the team-based organizations operated by Pharmaceutical Technologies in which analysts and formulators work closely together, a traditional request for analysis form is not required as the team inherently knows the status of its own samples. However, a laboratory information management system (LIMS) is used to track information on samples. This information is entered manually together with the tests required for the sample. Improvements needed include entry of sample information by bar code and specification/test information already available in the system enabling automatic generation.

(2) *Sampling.* This is critical in any analysis process and is generally performed manually, which can be time consuming and laborious. The sample size taken for the release testing of a drug product is generally very small compared with the batch size, e.g. 100 tablets from a 100 000 tablet batch. Furthermore, 30 tablets or less are typically used to provide the assay and content uniformity data to release the batch. Techniques that enable the analysis to be performed non-destructively without sampling in the manufacturing area either at-line or on-line are needed. This would enable a much larger and more representative sample size to be taken.

(3) *Analysis.* The release of a batch of drug product is generally dependent on limited in-process data and on testing of the finished product against a specification. Several improvements to this process are required. Firstly, more information on the quality of the product during the manufacturing process is required, and as discussed above, rapid non-destructive on-line techniques are required. The ultimate aim of this type of testing is to have sufficient in-process controls to enable a product to be released with little or no final product testing, i.e. parametric release. Secondly, the current time for automated tablet preparation (e.g. 15 min) is relatively long compared with the time for required HPLC or UV analysis, therefore more rapid sample preparation systems are required. Non-destructive methods would elegantly overcome this problem, but this will not be possible for all products. For example, for low-dose products, linking the separation with a sensitive detector (e.g. LC/MS) would offer a good solution. Finally, automatic results transfer to LIMS is typically available for outputs from HPLC systems. Links between robotic systems, e.g. the TPWII™ and MultiDose™ and the LIMS are also needed to provide easy transfer of sample information during the analysis. In the future, facile results transfer of the results from spectroscopic techniques, e.g. NIR and mass spectroscopy will also be needed.

(4) *Documentation.* The documentation of practical work from an analysis is time consuming and has resulted in analysts spending more time out of the laboratory. Typically, 15% of an analysts' time can be spent entering data into laboratory notebooks. In many laboratories, LIMS are used in an attempt to reduce the documentation burden, but the success and savings in time are largely dependent on the environment in which the system is used. In development, the rapidly changing project requirements tend to make the use of LIMS problematic such that information is recorded in laboratory notebooks, and the summary of results and associated references are transferred to LIMS. The net result from this process is that a proportion of information is duplicated as hardcopy and electronic files. In a commercial manufacturing QC, laboratories' work tends to be more predictable than in R&D, which has resulted in LIMS being implemented more successfully. Interfacing instruments including balances, HPLCs, UV spectrophotometers and associated robots to LIMS are needed to reduce the time spent writing-up in laboratory notebooks.

To reduce the wasted effort in transcribing information, electronic notebooks are being developed.

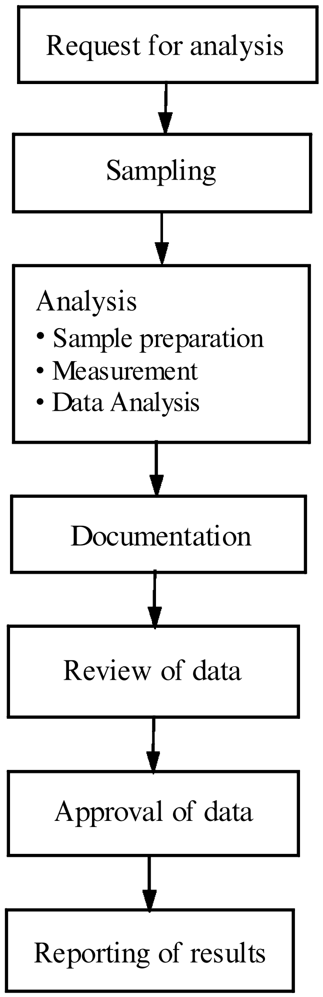

*Figure 2. Generic process for drug product analysis.*

The Collaborative Electronic Notebook Consortium Association (CENSA) is one such group that is moving the development of this technology forward [1]. A key element in the implementation of electronic information management is greater standardization of the working practices in laboratories. The increased use of robotics will help achieve this goal.

(5) *Review of data.* Reviewing of data is the backbone of the GMP process. Automated checking by the LIMS of key information in an analytical method should be possible. For example, LIMS could alert the analyst if the standard weights, sample weights, system suitability and the associated results are not within predetermined limits. This scenario is best suited to a standardized process operated by automated systems.

(6) *Approval of data.* For a product manufactured by a well-defined process, checking of results against a typical release specification can be undertaken automatically by LIMS. This has been performed successfully for many years in commercial manufacturing laboratories. Implementation in a development environment has not been widespread, which is probably due to the less well-defined processes and procedures for manufacturing and analysing the product.

(7) *Reporting of results.* In R&D, results are typically required for a number of main purposes. Firstly, to release product for clinical trials, which requires the generation of a Certificate of Analysis. Secondly, the generation of stability data to support product registration, which requires the data from several storage conditions tabulated in a form suitable for direct transfer into the regulatory document. The report generation capabilities of the LIMS system must be more flexible to provide data in the required form. Achieving this goal would dramatically decrease the time spent compiling and checking regulatory dossiers.

*Technology needs of manufacturing*

Rapid, reliable, easy-to-use and cost-effective methods are required by quality control (QC) laboratories in manufacturing. Methods based on robotics exhibit some of these desired attributes. The QC departments typically now require robotic methods for assay, content uniformity and dissolution methods from R&D prior to the manufacture of the qualification batches. In addition, for the validation batches, automated methods samples are needed for the analysis of powder samples taken at various time points from the mixers to demonstrate the homogeneity of the blending process. This work typically generates hundreds of powder samples for assay using TPWII™. More recently, QC laboratories are requesting NIR methods for assay, content uniformity and powder blend analysis. NIR offers rapid and non-destructive analysis compared to automated UV and HPLC technologies. This technique also has the added advantage of generating little waste compared with conventional techniques.

*Analytical techniques for the next millennium*

The discussion above indicates that there are emerging trends in analytical technologies that will increase productivity in pharmaceutical development, and these will be outlined below.

(1) *Parallel analytical processing.* To provide traditional analysis, e.g. HPLC at-line to monitor a manufacturing process, much faster systems are required. This could be achieved using fast HPLC systems; however, sample preparation instrumentation would have to be re-engineered to keep up with the separation speeds. The application of ultrasonic technology may help solve this problem. Parallel sample preparation and separation systems that enable the analysis to be completed in ~1 min will be required to compete with near-infrared.

(2) *Miniaturization.* Computer 'chip' technology is being developed to perform analyses. The so-called 'micro-TAS' (total analysis system) concept [2] combines sampling, separation, reaction and detection onto an integrated platform. This device is typically fabricated on silicon, glass or polymer wafers, on which micro minute features that are typically between 1 and 50 microns in size, are photoetched. The key benefits of this technology to pharmaceutical analysis are very rapid analysis time (seconds) and the small size of the equipment. This technology would lend itself to at-line analysis and to early development work when little compound is available. Sample preparation systems to match these devices are required before these devices are commercialized. The recent investment by Hewlett Packard in Caliper Technologies is an indication of the level of interest in 'chip' technology for analysis.

(3) *Non-invasive analysis.* Near-infrared [3] spectroscopy and Raman spectroscopy [4] are alternative techniques for the analysis of pharmaceutical products. No sample preparation is required, therefore analysis is rapid and lends the technologies to in-process testing as well as final product analysis. These technologies in most instances rely on building libraries of acceptable samples and comparing the unknown against this library. Linking these techniques to microscopes is now providing the capability of imaging materials with resolution down to about 10–20 microns. This technology is being used for troubleshooting; however, there may be utility for employing imaging in the routine testing of products and providing early warning of stability problems. Perkin Elmer have developed an automated system NIR imaging instrument. For highly potent low-dose drug products these techniques are generally not sensitive enough, and more traditional separation methods would be required for analysis.

(4) *Sensor technology.* Low levels of volatile residues in materials, e.g. packaging components are increasingly affecting the stability of drug products. Formaldehyde is a compound that has been shown to cross-link gelatin capsules and to cause stability problems with products. Neural nose technology has been used to monitor volatile residues in materials, particularly in the food industry. This technology uses electrically conducting organic polymers as sensors to monitor the aroma from materials together with neural networks to classify the aromas. Monitoring of raw materials and products on stability may be a rapid means of fingerprinting materials and predicting problems with volatile residues.

(5) *Fast sensitive and selective detectors*. The rapid development of LC/MS in terms of lower cost, ease of use and quantitative performance has meant that these systems will realize their potential to become routine detectors for HPLC. This may enable fast 'universal' liquid chromatography methods to be used in which the chromatography system is more of a means of sample introduction and any incomplete separations could be compensated by the mass spectrometer. This technology should lead to reduced time for method development and validation for quantitative methods, and provide more sensitive detection for degradation products, thus giving earlier warning of stability problems.

(6) *Informatics*. The technologies described above in (1)–(5) will provide more information on an increased number of samples. Experience to date with LIMS systems in a development environment indicates that significant improvements will be required to integrate information collection from both instruments and scientists (electronic laboratory notebooks). Once the information resides in the information management system, better reporting packages and links to regulatory management documents are necessary.

### Summary

The increased flow of development compounds from research and the needs of commercial manufacturing will require new technologies to be implemented. The trends in analytical techniques to meet these needs include: parallel analytical processing; miniaturization; non-invasive technologies; sensors; fast sensitive and selective detectors together with associated information management systems. New technologies are providing challenges and opportunities in pharmaceutical development. Successful application of automated methods and new analytical techniques requires a central team dedicated to the investigation and development of automated methods and non-invasive analytical techniques. Collaborations with other pharmaceutical companies and academic centres are providing more resources to introduce the technologies in a highly regulated pharmaceutical environment.

### Acknowledgements

Nigel North wishes to thank Les Brockhurst, Chris Banton, John Gostick, Kevin Smith and John Stanley for their help in developing new analytical techniques in Pharmaceutical Technologies.

### Trademarks

Zymate™, Zymark TPWII™ and Zymark MultiDose™ are registered trademarks of the Zymark Corporation.

### References

1. Collaborative Electronic Notebooks Consortium—www.censa.org
2. Ache, H. J., 1996, Chemical microanalytical systems: objectives and latest developments. *Fresenius J. Anal. Chem.*, **355**, 467–474.
3. Drennen, J. K., 1995, Near infrared spectroscopy: applications in the analysis of tablets and pharmaceutical dosage forms. *Applied Spectroscopy Reviews*, **30**, 139–174.
4. Niemczyk, T. M., 1998, Quantitative determination of Bucindolol concentration in intact gelatin capsules using Raman spectroscopy. *Anal. Chem.*, **70**, 2762–2765.

